# Emerging Perspectives on Dipeptide Repeat Proteins in C9ORF72 ALS/FTD

**DOI:** 10.3389/fncel.2021.637548

**Published:** 2021-02-18

**Authors:** Alexander Schmitz, João Pinheiro Marques, Irina Oertig, Niran Maharjan, Smita Saxena

**Affiliations:** ^1^Department of Neurology, Center for Experimental Neurology, Inselspital University Hospital, Bern, Switzerland; ^2^Department for BioMedical Research (DBMR), University of Bern, Bern, Switzerland; ^3^Graduate School for Cellular and Biomedical Sciences, University of Bern, Bern, Switzerland

**Keywords:** amyotrophic lateral scelerosis, C9ORF72 ALS/FTD, dipeptide repeat proteins (DPRs), RAN translation, neurodegeneration, motor neuron

## Abstract

The most common genetic cause of amyotrophic lateral sclerosis (ALS) and frontotemporal dementia (FTD) is a hexanucleotide expansion in the chromosome 9 open reading frame 72 gene (*C9ORF72*). This hexanucleotide expansion consists of GGGGCC (G_4_C_2_) repeats that have been implicated to lead to three main modes of disease pathology: loss of function of the C9ORF72 protein, the generation of RNA foci, and the production of dipeptide repeat proteins (DPRs) through repeat-associated non-AUG (RAN) translation. Five different DPRs are currently known to be formed: glycine–alanine (GA) and glycine–arginine (GR) from the sense strand, proline–alanine (PA), and proline–arginine (PR) from the antisense strand, and glycine–proline (GP) from both strands. The exact contribution of each DPR to disease pathology is currently under intense scrutiny and is still poorly understood. However, recent advances in both neuropathological and cellular studies have provided us with clues enabling us to better understand the effect of individual DPRs on disease pathogenesis. In this review, we compile the current knowledge of specific DPR involvement on disease development and highlight recent advances, such as the impact of arginine-rich DPRs on nucleolar protein quality control, the correlation of poly-GR with neurodegeneration, and the possible involvement of chimeric DPR species. Further, we discuss recent findings regarding the mechanisms of RAN translation, its modulators, and other promising therapeutic options.

## Introduction

Amyotrophic lateral sclerosis (ALS) is an adult-onset neurodegenerative disease characterized by the progressive degeneration of upper and lower motor neurons leading to hyperreflexia, spasticity, fasciculation, and muscle atrophy (Van Langenhove et al., 2012). Frontotemporal dementia (FTD) is another neurodegenerative disease that primarily affects the frontal and temporal lobes of the brain, resulting in progressive changes in behavior, personality, and/or speech (Van Langenhove et al., 2012; Strong et al., 2017). Based on overlapping clinical, genetic, and epidemiological data, ALS and FTD have recently been recognized as two ends of the same disease spectrum (Neumann et al., 2006; Lillo and Hodges, 2009). Approximately 15% of FTD patients show symptoms of ALS disease, whereas up to 50% of ALS patients have symptoms of FTD (Ng et al., 2014). In 2006, for the first time, both ALS and FTD were linked to chromosome 9 (Morita et al., 2006; Vance et al., 2006). Later in 2011, a hexanucleotide repeat expansion in the non-coding region of the *C9ORF72* gene was identified as a disease mutation common between both neurodegenerative diseases. This hexanucleotide repeat expansion, consisting of GGGGCC (G_4_C_2_) repeats, can be found in the first intron in the reading frame 72 of chromosome 9 (*C9ORF72*) in the non-coding region between exons 1 and 1b (DeJesus-Hernandez et al., 2011; Renton et al., 2011). Healthy individuals harbor less than 30 of these G_4_C_2_ repeats, while ALS/FTD patients with *C9ORF72* mutations carry 400 to several thousand G_4_C_2_ repeats (Taylor et al., 2016). Compared to the mutation in the *SOD1* gene, which was identified as the first causative gene for familial ALS, the expansion in the *C9ORF72* gene is twice as common in familial ALS patients (Renton et al., 2011).

**FIGURE 1:**
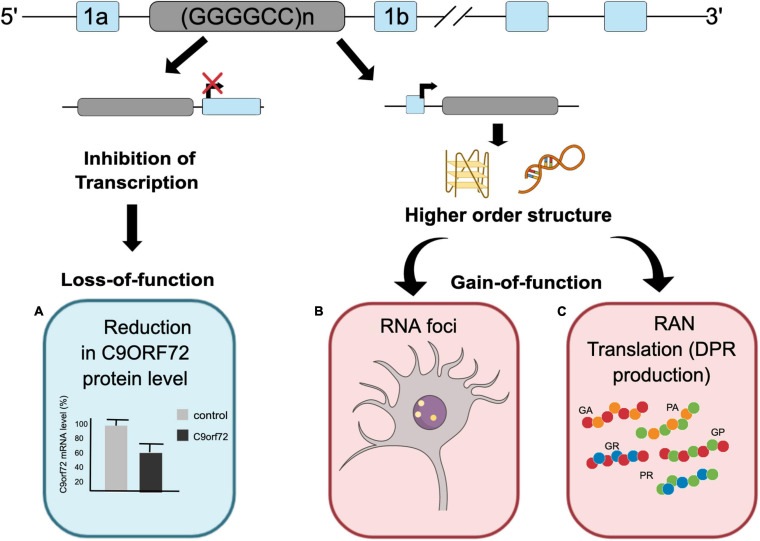
*C9ORF72* repeat associated disease mechanisms. The G_4_C_2_ repeat expansion can cause C9ORF72 ALS/FTD through three proposed mechanisms. **(A)** Reduction in *C9ORF72* protein levels. **(B)** RNA foci formation resulting in sequestration of different RNA binding proteins. **(C)** Accumulation of dipeptide repeat proteins (DPRs) generated through RAN translation.

To date, three different non-mutually exclusive mechanisms have been proposed to induce neurodegenerative changes through the G_4_C_2_ repeat expansion within the *C9ORF72* gene (Figure 1). The first mechanism involves the loss-of-function (LOF) of the C9ORF72 gene due to the hexanucleotide repeat expansion (reviewed by Braems et al., 2020). Evaluation of postmortem tissue of C9ORF72 ALS/FTD patients has identified a significant decrease in total C9ORF72 transcript levels (DeJesus-Hernandez et al., 2011; van Blitterswijk et al., 2015) and C9ORF72 protein levels (Waite et al., 2014; Frick et al., 2018) compared to healthy controls. Reduction of both mRNA transcript and protein levels was also observed in iPSC-derived motor neurons from C9ORF72 ALS/FTD patients,(Almeida et al., 2013; Shi et al., 2018) further supporting decrease in C9ORF72 level due to hexanucleotide repeat expansion. Although reduced levels of C9ORF72 protein cause motor neuron degeneration in *Caenorhabditis elegans* (Ciura et al., 2013) and zebrafish (Therrien et al., 2013), loss of C9ORF72 in mice did not elicit the ALS or FTD phenotype (Koppers et al., 2015). However, a reduction of C9ORF72 levels exacerbated neurodegeneration caused by the gain of toxicity of the repeat expansion (Zhu et al., 2020) and dipeptide repeat proteins (DPRs) (Shi et al., 2018). C9ORF72 protein functions as a RabGEF (Rab guanine nucleotide exchange factor) and is involved in vesicle trafficking and autophagy (Levine et al., 2013; Farg et al., 2014; Sullivan et al., 2016), implying that loss of C9ORF72 results in compromised autophagolysosomal clearance of toxic DPRs and misfolded proteins, which then leads to neuronal toxicity (Sellier et al., 2016; Shi et al., 2018).

The second pathogenic mechanism involves a gain-of-function (GOF) due to the formation of toxic RNA foci from repeat expansion transcripts (reviewed by McEachin et al., 2020b). RNA foci are a pathological hallmark of C9ORF72 ALS/FTD. Both sense and antisense RNA foci have been detected in multiple regions of central nervous system in C9ORF72 ALS/FTD patients and in different models of C9ORF72 ALS/FTD (DeJesus-Hernandez et al., 2011; Gendron et al., 2013; Batra and Lee, 2017). Various studies have shown that RNA foci are able to sequester functionally important RNA binding proteins (RBPs), potentially altering their localization and function (Xu et al., 2013; Zhang et al., 2015). Furthermore, RNA foci have been shown to correlate with the mislocalization of TDP-43 in both C9ORF72 ALS/FTD patients and mouse models (Chew et al., 2015; Aladesuyi Arogundade et al., 2019). In addition, the use of antisense oligonucleotides (ASOs) targeting the repeat expansion was able to mitigate C9ORF72 ALS/FTD-related pathology, highlighting the importance of RNA foci in C9ORF72 ALS/FTD pathogenesis (Donnelly et al., 2013; Lagier-Tourenne et al., 2013).

The third mechanism is a GOF due to the formation and accumulation of DPRs via repeat-associated non-AUG (RAN) translation of the hexanucleotide repeat sequences from both sense and antisense strands (reviewed by Freibaum and Taylor, 2017) (elaborated in this review). The involvement of both LOF and GOF in C9ORF72 ALS/FTD pathophysiology has been comprehensively investigated using different model systems. Although more than one of these mechanisms may contribute to C9ORF72 ALS/FTD pathology, the presence of DPRs in neurons implies that they likely play a crucial role in disease progression. However, the precise involvement of each DPR species in C9ORF72 ALS/FTD pathogenesis remains unresolved, further complicated by a recent finding implicating chimeric DPRs (cDPRs) in C9ORF72 ALS/FTD pathology. This review compiles the current knowledge about different DPRs and the recently identified cDPRs and discusses their relative contribution to C9ORF72 ALS/FTD pathogenesis.

## DPRs, the Gain of Toxic Function: Clues From Different Model Systems

DPR production is facilitated by non-canonical RAN translation, a mechanism first described in spinocerebellar ataxia type 8 (SCA 8) and myotonic dystrophy type 1 (DM1) (Zu et al., 2011). This finding was also quickly confirmed in many other microsatellite disorders and takes place in the absence of an AUG start codon and can occur in multiple reading frames. In C9ORF72 ALS/FTD, a hexanucleotide expansion of GGGGCC (G_4_C_2_) in the first intron of chromosome 9 produces five distinct DPRs from sense [poly-GA (glycine–alanine), poly-GP (glycine–proline), poly-GR (glycine–arginine) and antisense poly-GP (glycine–proline), poly-PR (proline–arginine), and poly-PA (proline–alanine)] (Mori et al., 2013; Zu et al., 2013) strands. These DPRs are amyloidogenic and accumulate in different parts of the central nervous system of C9ORF72 ALS/FTD patients (Ash et al., 2013; Mori et al., 2013; Zu et al., 2013). However, the pathogenic contribution of DPR-associated toxicity to disease progression is still unknown. In addition, the relative pathogenic contribution of each individual DPR remains unclear. Nevertheless, our understanding of DPR-related toxicity has been augmented through the use of codon-optimized constructs expressing each DPR independent of G_4_C_2_ repeats (May et al., 2014; Mizielinska et al., 2014; Jovicic et al., 2015). Although poly-GA is the most abundant DPR localizing in p62-positive inclusions in postmortem tissues from C9ORF72 ALS/FTD patients, multiple lines of evidence suggest that arginine-rich DPRs are the most toxic of the five DPRs in both *in vitro* and *in vivo* disease models (May et al., 2014; Mizielinska et al., 2014; Moens et al., 2019; Cook et al., 2020; Sun et al., 2020).

## Non-Arginine DPRs

Poly-GA is the most easily detected DPR in cytoplasmic inclusions (May et al., 2014; Zhang et al., 2014) not only due to its high translation efficiency, but also due to the predicted structural properties of the poly-GA peptide (Chang et al., 2016). Indeed, evidence suggests that poly-GA tends to aggregate into amyloid-like fibrils, which form a parallel β-sheet structure (Chang et al., 2016; Edbauer and Haass, 2016; Brasseur et al., 2020). Due to the biophysical similarities between poly-GA aggregates and Alzheimer’s disease–associated amyloid-beta peptides, it is proposed that poly-GA DPRs may trigger TDP-43 pathology in C9ORF72 ALS/FTD in the same manner as the amyloid-beta neurodegeneration cascade in Alzheimer’s disease (Edbauer and Haass, 2016). Similar to poly-GA toxicity observed in cell cultures, poly-GA–overexpressing mice display motor and cognitive deficits combined with cerebellar atrophy, astrogliosis, and TDP-43 pathology (Chew et al., 2015; Chang et al., 2016; Khosravi et al., 2020). Furthermore, consistent with its biophysical properties, poly-GA induces cellular toxicity by sequestering different proteins such as Unc119, which functions to regulate axonal protein trafficking and synaptic signal transduction (Maduro et al., 2000), or by directly inhibiting proteasomal activity. May et al. (2014) showed that sequestration of Unc119 by poly–GA inhibited its function and contributed to selective neuronal vulnerability in C9ORF72 ALS/FTD. Interestingly, this study reported that proteasomal proteins were not sequestered by poly-GA and that their activity was not affected *in vitro* (May et al., 2014). Contrarily, other groups have shown that poly-GA directly associates with and inhibits the proteasome, thereby promoting TDP-43 pathology (Zhang et al., 2014; Khosravi et al., 2020). The proteasomal subunit PSMC4 was found to colocalize with GA aggregates in poly-GA overexpressing mice and in C9ORF72 ALS/FTD patient tissue (Khosravi et al., 2020). Despite the binding partners of poly-GA remaining unclear, other studies report that poly-GA promotes endoplasmic reticulum (ER) stress and activation of caspase 3–related apoptotic pathways, as well as proteasome inhibition *in vitro* (May et al., 2014; Zhang et al., 2014; Khosravi et al., 2020), leading to reduced dendritic branching in neuronal cultures overexpressing poly-GA compared to control cultures. Further evidence of poly-GA’s proteasomal involvement can be seen by the overexpression of the proteasome protein HR3B, which partially rescues poly-GA–induced toxicity (Chew et al., 2015). In addition, the promotion of proteasome activity through rolipram treatment or the overexpression of the proteasome protein PSMD11 rescued poly-GA aggregation and TDP-43 pathology *in vitro* (Khosravi et al., 2020).

Besides protein sequestration and proteasomal inhibition, poly-GA expression was found to decrease the efficiency of DNA double-strand break repair mechanisms, specifically impacting non-homologous end joining, single-strand annealing, and microhomology-mediated end joining processes (Andrade et al., 2020). Furthermore, mobile poly-GA aggregates can be found in the axons and dendrites of primary cortical and motor neurons overexpressing the poly-GA DPR (Jensen et al., 2020). Evidence indicates that neurons with poly-GA aggregates have increased Ca^2+^ influx in response to an external stimulus; nevertheless, the synaptic release is abrogated in these neurons. This study proposes that poly-GA aggregates lead to synaptic dysfunction by reducing the levels of synaptic vesicle–associated protein 2 (SV2), an essential component of synaptic release machinery that forms complexes with other vesicle components such as synaptophysin (Mutch et al., 2011). Indeed, it was confirmed that SV2 levels are reduced in induced pluripotent stem cell (iPSC) lines of C9ORF72 ALS patients and in the spinal cord and neuromuscular junctions of poly-GA–overexpressing transgenic mice. Moreover, restoring the levels of SV2 in primary cortical and motor neurons rescues synaptic function and poly-GA–induced cellular toxicity (Mutch et al., 2011). Although the mechanisms by which poly-GA induces toxicity are still disputed and potential novel pathogenic mechanisms are still being uncovered, poly-GA DPRs appear to be less toxic than arginine-containing DPRs (Mizielinska et al., 2014; Wen et al., 2014; Freibaum et al., 2015). Hence, despite being the most readily detected DPR in inclusions, it is not clear whether poly-GA is toxic at physiologically relevant levels.

The DPRs poly-GP, poly-PA, and poly-GA are all uncharged; however, unlike poly-GA, the two former DPRs have a flexible coil structure and thus are unable to aggregate by themselves (Lee et al., 2016; Freibaum and Taylor, 2017). Consistent with the predicted biophysical proteins of such structures, these DPRs interact with fewer intracellular proteins when compared to other DPR species (Lee et al., 2016), suggesting that poly-GP and poly-PA are probably the least toxic species. Indeed, these DPRs when expressed in *Drosophila* models were not toxic (Mizielinska et al., 2014; Wen et al., 2014; Freibaum et al., 2015; Lee et al., 2016). Contrarily, Yamakawa et al. (2015) found that, *in vitro*, poly-GP increases cell death in the presence of the proteasome inhibitor MG-132 and inhibits degradation of the reporter construct Ub-G76V-GFP, which was used to assay the activity of the ubiquitin-proteasome system. Poly-GP levels detected in the cerebrospinal fluid (CSF) of both asymptomatic *C9ORF72* mutation carriers and symptomatic cases revealed that poly-GP concentration in the CSF is stable during disease, and its levels do not correlate with disease onset and clinical scores. Despite this, CSF poly-GP levels have the potential to be a useful marker to distinguish *C9ORF72*-associated disease from other neurodegenerative diseases and may aid in the identification of *C9ORF72* mutation carriers (Gendron et al., 2017; Lehmer et al., 2017). Additionally, an ASO targeting the G_4_C_2_ transcript in a cell and a mouse model of C9ORF72 ALS/FTD resulted in decreased poly-GP levels and inhibited DPR-associated toxic effects. This suggests that CSF poly-GP levels could be reliably used to assess the effectiveness of G_4_C_2_-RNA therapies as a surrogate way to measure total DPR load (Gendron et al., 2017).

## Arginine-Rich DPRs

Our current concept of toxicity caused by the DPRs poly-GR and poly-PR has evolved dramatically in recent years, with the focus shifting toward toxic mechanisms elicited by their highly interactive nature. Many disease models that focus on the overexpression of different DPR species have shown that poly-GR and poly-PR are toxic to HEK293T cells, primary neuronal cultures, and iPSC derived cortical and motor neurons (Wen et al., 2014; Tao et al., 2015; Lee et al., 2016), while the expression of the other DPRs is not toxic (Mizielinska et al., 2014; Wen et al., 2014; Freibaum et al., 2015; Lee et al., 2016). This can partly be explained by their unique positive charge and high polarity, conferred by arginine. Poly-GR and poly-PR are noticeably more hydrophilic and less prone to aggregation than poly-GA (Kwon et al., 2014). Both DPRs can also be easily transported into the nucleus, as they possibly mimic nuclear localization signal domains that tend to be rich in arginine (Kwon et al., 2014). Indeed, in a landmark study by Kwon et al. (2014), it was shown that exposing cells to synthetic poly-PR and poly-GR enabled the arginine-rich DPRs to enter the nucleus, bind to nuclear puncta, disrupt ribosomal RNA (rRNA) production, and drastically reduce cell viability in U2OS cells. They thus offered an alternative explanation of poly-GR/PR–generated toxicity, as previous studies focused on the postulated toxicity of mainly cytoplasmic poly-GR and poly-PR aggregates, both *in vitro* and in patient tissue (Ash et al., 2013; Mori et al., 2013; Zu et al., 2013). Separately in 2015, multiple studies were published focusing on the low complexity sequence domains (LCDs) of RBPs such as hnRNPA1 (Molliex et al., 2015) and FUS (Murakami et al., 2015; Patel et al., 2015) and demonstrated both their ability in mediating the liquid–liquid phase separation (LLPS) of stress granules and their propensity, if mutated, to undergo irreversible liquid–solid phase transitions. The concepts of poly-GR– and poly-PR–mediated toxicity and RBPs capable of facilitating LLPS were then elegantly combined to show that arginine containing DPRs are able to interact with the LCDs of RBPs and significantly alter the dynamics of LLPS of multiple membrane-less organelles (Lee et al., 2016). These findings offer a mechanistic framework to account for disrupted nucleolar transport and nuclear pore function in C9ORF72 ALS/FTD (Freibaum et al., 2015). Furthermore, these observations opened up the exploration of diverse mechanisms by which arginine containing DPRs are able to perturb the physiological LLPS of different membrane-less organelles, such as stress granules (Boeynaems et al., 2017; Zhang et al., 2018) and the nucleolus (Lee et al., 2016; White et al., 2019) in C9ORF72 ALS/FTD. Indeed, White et al. (2019) were able to confirm the involvement of poly-GR and poly-PR in the nucleolus and elaborated on poly-PR’s ability to bind and change the biophysical properties of the LCD containing nucleophosmin1 (NPM1) and modulate NPM1’s ability to undergo LLPS with its physiological binding partners containing arginine-rich motifs. While purified poly-PR in low concentrations was able to induce the LLPS of NPM1, it caused droplet dissolution at higher concentrations. Additionally, poly-PR sequestered NPM1 into large soluble poly-PR–bound complexes, attributed to its ability to inhibit LLPS. In HeLa cells, a delocalization of NPM1 out of the nucleolus was observed, confirming a phenomenon seen in a previously published study (Farg et al., 2017). This delocalization could not be observed with NPM1 and its physiological binding partners capable of inducing the LLPS of NPM1. As NPM1 is heavily involved in ribosome biogenesis and transport, among many other cellular processes, DPR binding and displacing NPM1 and, hence interfering with NPM1’s ability to fulfill its physiological functions, could significantly contribute to DPR-mediated toxicity.

The downstream consequences of poly-GR and poly-PR disrupting NPM1 have not yet been fully established. One factor is that our current understanding of NPM1’s wide functional repertoire is still not complete. NPM1 has recently been implicated to also play a role as an integral component of the nucleolus’s protein quality control machinery (Frottin et al., 2019). It was shown that exposing cells to heat stress caused potentially highly interactive and aggregation-prone misfolded nuclear proteins upon entering the nucleus to become immobile by reversibly binding to NPM1 (Frottin et al., 2019). Upon stress resolution, the stored proteins underwent Hsp70-guided refolding. Both extended intervals of induced stress and expression of poly-PR compromised the nucleolus’s capacity for protein quality control (Frottin et al., 2019) and resulted in the formation of pathological aggregates of misfolded proteins in the nucleoplasm that sequestered neighboring proteins. Poly-PR may also diminish NPM1’s role in DNA repair, resulting in a less efficient mechanism of double-strand break repair (Andrade et al., 2020), which most likely leads to increased DNA damage and cell death (Farg et al., 2017).

Nucleolar toxicity of poly-PR and poly-GR, and by extension their cellular toxicity in general, appears to arise from their disruption of different vital physiological processes and not by their aggregation. Although the expression of their toxic nature seems ostensibly straightforward and to originate in their high interactivity and ability to interrupt LLPS, their nucleolar binding partners and binding effects are multifaceted and complex. These include the inhibition of protein translation through the binding of the translation initiation factor eIF3η (Zhang et al., 2018), dysfunctional rRNA processing and impairment of ribosome biogenesis through the binding of ribosomal subunits (Tao et al., 2015; Suzuki et al., 2018), and most recently disruption of the nucleolar protein quality machinery through the interaction with NPM1.

Despite evidence showing the toxic effects of arginine-containing DPRs, especially in the nucleus, the relative contribution of poly-GR and poly-PR to human disease is still unknown. Furthermore, although these DPRs share approximately 40% of their interactome with each other, it is likely that the sense and antisense strands of the *C9ORF72* hexanucleotide repeat are not translated at comparable rates (Lee et al., 2016). Additionally, poly-GR and poly-PR DPRs have been found to localize in different regions of the cell. While poly-PR tends to localize in the nucleolus, poly-GR concentrates mostly in the cytoplasm, which could indicate that poly-GR plays a more important role in disrupting stress granule dynamics (Wen et al., 2014; Lee et al., 2016). Furthermore, cytoplasmic poly-GR was found to localize to mitochondria where it associates with mitochondrial ribosomal proteins, thereby inducing oxidative stress (Lopez-Gonzalez et al., 2016).

Nevertheless, the development of mammalian models to study poly-GR and poly-PR DPRs is essential to understand how these DPRs contribute to disease manifestation. A recently developed mouse model expressing *C9ORF72* with repeat expansions via a bacterial artificial chromosome (BAC) displays motor impairments and neurodegenerative features of ALS/FTD seemingly associated with detectable poly-PR expression (Liu et al., 2016). Despite this, it is unknown whether poly-PR is the most toxic DPR species and how it promotes disease phenotypes in this model. It was recently shown that arginine-containing DPRs are able to directly elicit defective intracellular trafficking of different cargos including mitochondria and RNA granules by interacting with microtubule and motor proteins (Fumagalli et al., 2019). However, more evidence is required to determine the exact roles the arginine-containing DPRs poly-GR and poly-PR play in the development of the ALS phenotype *in vivo*.

## DPR Pathology and Its Correlation to Neuronal Toxicity: Current Clues From Human Patients

One major challenge facing C9ORF72 ALS/FTD research today is reconciling the apparent discrepancy between the pattern of DPR pathology seen in human postmortem tissue and in different animal and *in vitro* models. The first human pathological reports identified cardinal features such as the presence of phosphorylated TDP-43 inclusions (Murray et al., 2011; Boeve et al., 2012; Cooper-Knock et al., 2012; Gijselinck et al., 2012; Solomon et al., 2018) as well as p62 and ubiquitin-positive, but TDP-43–negative, neuronal cytoplasmic inclusions, in human C9ORF72 ALS/FTD tissue (Al-Sarraj et al., 2011; Boxer et al., 2011; Mahoney et al., 2012; Bigio et al., 2013). After the discovery of RAN translation in different microsatellite disorders (Zu et al., 2011), RAN-translated proteins were also confirmed in C9ORF72 ALS/FTD via positive immunostaining for different DPR species, throughout the central nervous system in C9ORF72 patient neurons (Ash et al., 2013; Mori et al., 2013; Zu et al., 2013). Multiple clinicopathological studies hereafter were published, with significant differences concerning methodology, antibodies used, and regions of the nervous system extensively tested (Al-Sarraj et al., 2011; Cooper-Knock et al., 2012; Mahoney et al., 2012; Troakes et al., 2012). Further extensive characterization of the anatomical localization of individual DPR species was compiled in a systematic neuropathological review by Schipper et al. (2016), where authors appraised 42 studies surveying a total of 262 patients. The analysis revealed that DPRs were most commonly found in the frontal lobe (97.2%), hippocampus (97.1%), temporal lobe (92.5%), and cerebellum (90.9%) and only to a lesser extent in the spinal cord (49.8%) in C9ORF72 positive patients (Schipper et al., 2016). The question thereafter emerged: To what extent does the production of DPRs confer neurodegeneration *in vivo*, and subsequently, which DPR species contributes the most to the toxicity process? In a study by Davidson et al. (2014), analysis of their pathological data revealed that neither the anatomical location nor the amount of poly-GA correlated with the clinical phenotype or the extent of TDP-43 pathology. Similar observations were echoed by another pathological study that also could not detect a clear correlation of any DPR inclusion with neurodegeneration (Mackenzie et al., 2015). In contrast, cellular models and animal models seem to indicate that the arginine-rich DPRs are quite toxic (Wen et al., 2014; Tao et al., 2015; Lee et al., 2016) with poly-GA exhibiting less fulminant toxicity or none at all (Wen et al., 2014). It remains to be fully explored why no correlation between the degree of DPR pathology and neurodegeneration could be seen, when different C9ORF72 disease models strongly suggest some contribution of DPRs to disease burden. Study methodology of the pathological tissue may play a crucial role, demonstrated by a study that stratified the brain in disease-related (frontal cortex, motor cortex, anterior horn of the spinal cord) and disease-unrelated (parietal cortex, occipital cortex, posterior horn of the spinal cord) regions. Such an approach revealed that the abundance of cytoplasmic poly-GR robustly correlated with cellular and neuroanatomical pathology (Saberi et al., 2018). This indeed was somewhat confirmed by another study a year later, wherein a strong correlation was found between poly-GR density and neurodegeneration in the frontal cortex using quantitative digital microscopic methods (Sakae et al., 2018). In contrast, a recent study identified an association of DPR inclusions in muscle, mostly poly-GA and poly-GP, but not poly-GR with muscle atrophy in C9ORF72 ALS patients implying that DPRs in muscles may also contribute to ALS pathology and that DPR pathology is not exclusive to neurons (Cykowski et al., 2019). However, different studies have implicated poly-GR as the DPR species that is associated with neurodegeneration (at least in disease-affected areas) in postmortem C9ORF72 ALS tissue. Nevertheless, definitive consensus regarding the DPR species that provides the most robust correlation with neurodegeneration is still missing (Table 1).

**TABLE 1:** Summarizing the detection of DPRs in different human brain regions according to various neuropathological studies examining postmortem brain tissue.

Studies	Frontal lobe	Temporal lobe	Basal ganglia	Brainstem	Cerebellum	Spinal cord	Skeletal muscle
Mori et al. (2013)	N/A	GA, GR, GP	N/A	N/A	GA, GR, GP	N/A	N/A
Gendron et al. (2013)	PA, PR, GP	PA, PR, GP	N/A	PA, PR, GP	PA, PR, GP	N/A	N/A
Mann et al. (2014)	N/A	GA, GP, GR, PR, PA	N/A	N/A	GA, GP, GR, PR, PA	N/A	N/A
Zu et al. (2013)	GA, GP, GR, PA, PR	GA, GP, GR, PA, PR	N/A	N/A	N/A	GP, GA(–), GR(–), PR(–), PA(–)	N/A
Mackenzie et al. (2013)*	GA	GA	GA	GA	GA	GA	N/A
Gomez-Deza et al. (2015)	N/A	N/A	N/A	N/A	N/A	GA, GR, GP, PA, PR	N/A
Mackenzie et al. (2015)	GA, GP, GR, PA, PR	N/A	N/A	N/A	GA, GP, GR, PA, PR	GA, GR, GP, PA, PR	N/A
Schludi et al. (2015)	GA, GR, GP, PR	GA, GR, GP, PR	GA, GR, GP	GA, GR, GP	GA, GR, GP, PR	GA, GR, GP, PR(–)	N/A
Davidson et al. (2014)	GA, GP, GR	GA, GP, GR, PA (–), PR (–)	N/A	N/A	GA, GP, GR, PA(–), PR(–)	GA, GP, PR, PA**	N/A
Saberi et al. (2018)	GA, GP, GR, PA, PR	GA, GP, GR, PA, PR	N/A	N/A	GA, GP, GR, PA, PR	GA, GP, GR, PA(–), PR(–)	N/A
Cykowski et al. (2019)	N/A	GA	N/A	N/A	N/A	N/A	GA, GP, GR (–)
Sakae et al. (2018)	GA, GP, GR	GA, GP, GR	N/A	N/A	N/A	N/A	N/A

(–) indicates that the DPR was tested for but not detected. *Poly-GA was the only DPR tested using a monoclonal poly-GA antibody. **Positive poly-PR and poly-PA staining was found also in the control anterior horn cells.

Despite p62-positive cytoplasmic aggregates being the most prominent pathological hallmark in the frontal cortex of *C9ORF72*-associated diseased patients, some postmortem studies describe the presence of p62-negative paranucleolar bodies (Schludi et al., 2015) and poly-PR DPR localization to intranuclear aggregates (Wen et al., 2014), which suggests that DPR-positive inclusions also localize to the nucleolus in *C9ORF72*-associated disease. Furthermore, various studies indicate that DPR pathology predates TDP-43 pathology (Baborie et al., 2015; Vatsavayai et al., 2016), which suggests that DPR-related toxicity may be responsible for the early progression of the disease.

In addition, while clinicopathological studies are vital in contextualizing *in vitro* and animal models of disease, a bias may be present when analyzing tissue from already deceased individuals, as a pathological still image of the end-stage disease may not be accurately portraying the chain of events leading to neurodegeneration. Information is lost concerning neurons that have already perished before pathological analysis, and some DPR-positive neuronal inclusions observed may be mere bystanders and may not have been central to pathological neuronal decline. Furthermore, the pathological contribution of soluble DPRs may be underrepresented, as soluble protein species can be difficult to observe and analyze in fixed tissue. In line with this, Quaegebeur et al. (2020) used protein fractionation and immunoassay to quantify both soluble and insoluble DPRs in brain homogenates of FTD patients with *C9ORF72* repeats and found that clinically affected areas have less soluble DPRs compared to the cerebellum, which is unaffected in C9ORF72 FTD. The exact role DPRs play in shaping the course of events leading to neurodegeneration thus remains elusive and cannot be satisfyingly answered by solely analyzing postmortem tissue from C9ORF72 ALS/FTD patients.

## Insights Into Ran Translation and Production of DPRs

**FIGURE 2:**
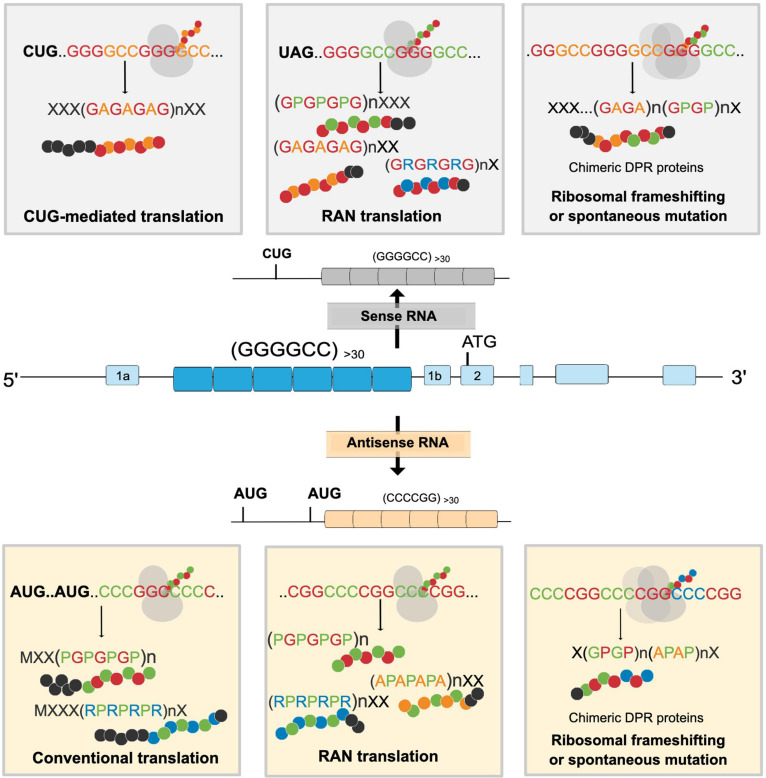
Proposed mechanism for the formation of different dipeptide repeat proteins (DPRs) through the G_4_C_2_ repeat expansion in the *C9ORF72* gene. Both sense and antisense strands can be translated through CUG-initiated, conventional AUG, and RAN mediated translation resulting in the formation of different DPRs. cDPRs are proposed to occur because of ribosomal frameshifting or spontaneous mutations in the G_4_C_2_ repeat expansion. X denotes aminoacids present in N- or C- terminus of DPRs.

Considering the increasing importance RAN-translated proteins have in C9ORF72 ALS/FTD pathology, recent studies have also focused on understanding the mechanisms governing G_4_C_2_-mediated RAN translation. Translation of G_4_C_2_ occurs in both a 5′ cap-dependent manner (Green et al., 2017) and/or cap-independent manner (Cheng et al., 2018) (albeit less efficiently) and can exhibit many other properties of canonical translation initiation such as involvement of methionyl-initiator tRNA (tRNA_i_^Met^) and translation initiation factors (eIF4E, eIF4G, and eIF4A) (Tabet et al., 2018). In the sense strand, DPR production is likely initiated through a near-cognate CUG codon present 24 nucleotides upstream of the G_4_C_2_ repeat expansion in the GA reading frame and in an optimal Kozak sequence. Poly-GA is therefore mostly translated through a conventional ribosomal scanning mechanism (Tabet et al., 2018; Figure 2). In contrast, poly-GP, which is in the + 2 reading frame, has a UAG stop codon in its reading frame located at the beginning of the G_4_C_2_ repeat expansion, implying that production of poly-GP is enabled either directly through RAN translation initiation within the repeat itself or indirectly by translation initiation in the poly-GA reading frame followed by a ribosomal frameshift to the poly-GP frame. Indeed, mutations in the CUG codon prevented the formation of all three DPRs encoded from the sense strand [poly-(GA, GP, GR)] and therefore gave support to ribosomal frameshifting mechanisms for the production of poly-GP and poly-GR from different reading frames (Tabet et al., 2018). Furthermore, translation efficiency of GA was the highest as the production of the other DPRs requires at least one frameshifting event, thereby confirming the results of previous clinicopathological studies, wherein poly-GA has been found to be the most abundant DPR (Mackenzie et al., 2015; Lee et al., 2017).

Although the DPRs produced from G_2_C_4_ antisense transcripts, poly-PA, poly-PR, and poly-GP are present in postmortem C9ORF72 ALS/FTD tissue samples; the exact translation mechanisms for these antisense transcripts are still unknown. These antisense DPRs are speculated to be translated from two open reading frames encoding for poly-PR and poly-GP, respectively (Figure 2). However, the presence of poly-PR and poly-GP even in the absence of AUG initiation sites implicates the expression of antisense DPRs via RAN translation (Gendron et al., 2013; Zu et al., 2013) as well. This thus opens the possibility for the formation of three RAN-translated antisense DPRs and two putative AUG-initiated antisense DPRs. Poly-GP is translated from both strands; however, poly-GP translated from the antisense strand is not identical to that generated from the sense strand. In the antisense strand, the poly-GP repeat has a stop codon immediately after the repeat while the sense strand contains a unique C-terminal sequence. By using an antibody against the unique C-terminal sequence and poly-GP domain, Zu et al. (2013) were able to distinguish poly-GP generated from the sense and antisense strand. They found that the majority of poly-GP inclusions in neurons were in fact produced from the antisense strands (Zu et al., 2013). This can be explained by the finding that in the antisense strand, poly-GP is translated via AUG-initiated translation as well as RAN translation, whereas in the sense strand poly-GP is translated through RAN translation (Zu et al., 2013; Tabet et al., 2018). Intriguingly, the C-terminal region of RAN-translated proteins can influence their cellular distribution and relative toxicity (He et al., 2020), suggesting that poly-GP produced from either the sense or the antisense strand could have different biochemical properties and interacting partners.

## Cellular Stress and RAN Translation: A Possible Therapeutic Target

Different cellular stresses, including oxidative stress and ER stress, induce the integrated stress response (ISR), a process vital for both cell survival and apoptosis. The ISR is induced primarily via different kinases such as GCN2 (amino acid starvation), PERK (ER stress), HRI (oxidative stress), and PKR (DNA damage) that phosphorylate the α subunit of eIF2, an initiation factor that mediates the binding of tRNA_i_^Met^ to the 40 s subunit of the ribosome, creating a ternary complex with GTP (Krishnamoorthy et al., 2001). The complex so formed then binds to the AUG start codon, leading to GTP hydrolysis requiring eIF2B, a guanine nucleotide exchange factor, to substitute GDP with GTP, in order to recommence translation initiation (Webb and Proud, 1997). eIF2B is only able to replace GDP with GTP in its unphosphorylated state. eIF2B binds with a higher affinity to phosphorylated eIF2α (p-eIF2α) and, once bound to p-eIF2α, has its ability to replace GDP with GTP suppressed (Nika et al., 2001) and thus arrests global protein synthesis. Activation of the ISR, therefore, ultimately leads to the cessation of conventional cap-dependent protein translation via the phosphorylation of eIF2α, thereby drastically reducing protein synthesis. However, cap-independent translation can still persist, resulting in expression of selective proteins that are necessary for cell survival and recovery (Bhattacharyya et al., 2006; Zhou et al., 2008; Lawless et al., 2009).

Cellular stress is a prominent feature of C9ORF72 ALS/FTD (McEwen et al., 2005; Dafinca et al., 2016; Lopez-Gonzalez et al., 2016; Kramer et al., 2018; Westergard et al., 2019). RAN translation in C9ORF72 ALS/FTD is impervious to the inhibitory protein synthesis effects of eIF2α phosphorylation and is, in fact, selectively enhanced by the ISR (Green et al., 2017). In addition, initiation of RAN translation is essentially dependent on eIF2α phosphorylation under conditions of cellular stress (Green et al., 2017; Cheng et al., 2018; Sonobe et al., 2018). However, it is still unclear which initiation factors are used in states of cellular stress, and there is tentative evidence that alternative initiation factors such as eIF5B, eIF2D, and eIF2A may be partially involved (Starck et al., 2016), at least in the translation of poly-GA (Sonobe et al., 2018). In C9ORF72 ALS/FTD, the ISR can be engaged by different DPRs primarily by their ability to elicit ER stress (Dafinca et al., 2016; Kramer et al., 2018) and oxidative stress (Lopez-Gonzalez et al., 2016), but can also be induced by excitotoxic stress and repeated neuronal depolarization (Westergard et al., 2019). In addition, the formation of stress granules is also known to be eIF2α dependent (McEwen et al., 2005), and this dependency was shown to also apply to stress granules formed by G_4_C_2_ repeats. Moreover, *C9ORF72* protein itself is also known to play an important role in stress granule dynamics and has been implicated as a regulator of the cellular stress response (Maharjan et al., 2017; Chitiprolu et al., 2018). DPRs (and G_4_C_2_ RNA) can not only induce the formation of stress granules but also enhance RAN translation and thus their own production, while inhibiting global protein synthesis, resulting in a feed-forward mechanism mediated by the phosphorylation of eIF2α. Paradoxically, simultaneous inhibition of global protein synthesis might indeed also apply to proteins involved in the degradation of DPRs, thereby further increasing total DPR load. However, it is yet to be fully explored how exactly phosphorylation of eIF2α affects the alternative translation initiation mechanisms that are proposed to enhance RAN translation, and which form of stress and kinases involved have the most profound effect on RAN translation.

One cellular stress pathway that has recently received attention is the activation of the PKR pathway. Although it was initially posited two decades ago that CUG repeat expansions could form RNA hairpins that can then activate the PKR (Tian et al., 2000), definitive evidence implicating G_4_C_2_ repeats in forming RNA hairpins and activating the PKR has been lacking. However, recent studies show that the PKR pathway regulates RAN translation both dependent and independent of eIF2α phosphorylation, as inhibition of the PKR pathway was able to reduce RAN-translated poly-GA and poly-GP significantly more than inhibition of p-eIF2α alone in HEK293T cells (Zu et al., 2020). Metformin has shown remarkable therapeutic promise in its ability to inhibit the PKR pathway and promote the reduction of poly-GA and poly-GP levels in C9-BAC mice (Zu et al., 2020). However, it remains undetermined by which mechanism the PKR pathway can independently induce RAN translation and to what extent metformin and other PKR inhibitors would be viable therapeutic options for patients with C9ORF72 ALS/FTD.

The search for RAN translation modulators has also been extended to genetic modifiers. Recently, it was shown that yeast is also able to undergo RAN translation, and via a genetic screen of different yeast mutants, it was discovered that both deleting the gene RPS25A in yeast and targeting the mammal homolog, RPS25, with an ASO were able to reduce poly-GP levels by 50%. Importantly, this process did not reduce AUG-mediated global protein synthesis (Yamada et al., 2019). Yuva-Aydemir et al. (2019) sought out another approach by demonstrating that the knockout of the translation elongation factor AFF2/FMR2 rescues axonal degeneration and TDP-43 pathology by decreasing the expression of mutant *C9ORF72* allele and consequently, reduced the levels of RNA foci and DPRs in iPSC-derived cortical neurons from C9ORF72 patients. Another possible method may involve targeting transcriptional regulators of the hexanucleotide repeat expansion. Transcription of the G_4_C_2_ repeat in C9ORF72 ALS/FTD is regulated by the PAF1 complex, which functions as a transcriptional regulator of RNA polymerase II. Evidence suggests that in *Drosophila* PAF1 complex components have a higher affinity for long toxic repeat expansions rather than shorter non-toxic expansions. Furthermore, PAF1 is upregulated in cells derived from C9ORF72 patients and following G_4_C_2_ repeat expression in *Drosophila* and mice (Goodman et al., 2019).

More extensive studies will be needed to determine how effective both inhibiting DPR-induced stress and/or targeting different genes important for RAN translation with ASOs will be, and importantly, which potential side effects these treatment options may have. While these results sound promising, other therapeutic targets might revolve around the mitigation of DPR load by directly targeting the individual species. Our current understanding, however, of the exact nature and effects of these individual DPR species is highly dynamic as researchers try to unravel the novel mechanisms by which they induce toxicity.

## Future Directions and Conclusion

Although it is accepted that specific DPR species have toxic effects in cellular and animal models, their precise contribution to *C9ORF72*-associated disease progression is still disputed. Naturally, C9ORF72 ALS/FTD patients are likely to express all DPRs and not only a single specific DPR. Thus, it is essential to understand how individual DPRs may interact with each other at physiologically relevant levels to promote disease pathology. Nevertheless, this is currently an underexplored area of the *C9ORF72*-associated ALS/FTD field.

Recent evidence indicates that DPRs might not be translated as single dipeptide entities and could instead be translated in combination due to ribosomal frameshifting (McEachin et al., 2020a). McEachin et al. (2020a) coined the term “chimeric DPRs” to describe the nature of these putative DPRs. They showed that SCA36, an ataxic disorder caused by an intronic TG3C2 hexanucleotide expansion, also undergoes RAN translation to produce different DPRs, including the DPRs poly-GP and poly-PR. As poly-GP also is a product of *C9ORF72* RAN translation, poly-GP generated in SCA36 therefore was expected to possess similar solubility characteristics to poly-GP from C9ORF72 ALS/FTD. Counterintuitively, poly-GP was shown to be diffusely expressed in SCA36 neurons, contrasting the presence of perinuclear inclusions of poly-GP in C9ORF72 ALS/FTD neurons. This discrepancy was reconciled with the idea that poly-GA can mediate the aggregation of poly-GP and supported by the finding that 90% of poly-GP colocalized with poly-GA in postmortem C9ORF72 ALS/FTD tissue and C9-BAC mice. Similarly, transfection with GA:GP chimeric constructs was able to induce the formation of GA:GP inclusions, whereas cotransfection with a poly-GA and poly-GP construct did not induce colocalization (McEachin et al., 2020a), further strengthening the hypothesis that aggregation-prone poly-GA contributes to the poly-GP pathology seen in *C9ORF72* ALS/FTD. New insights into the mechanisms modulating RAN translation, especially ribosomal frameshifting, and the occurrence of repeat interruptions in G_4_C_2_ transcripts indicated by using long-read sequencing technologies (Ebbert et al., 2018) lend further mechanistic support to the possibility of the translation of cDPR species (Figure 2). However, it still needs to be unequivocally proven that indeed cDPR species are translated in C9ORF72 ALS/FTD patients and then further determined to what extent these species may contribute to the total disease burden.

Several other studies have demonstrated the interaction between different DPRs. Overexpressing different DPRs in Neuro2A revealed that poly-GR and poly-PR DPRs might recruit poly-GA and poly-GP DPRs into cytoplasmic inclusions (Yamakawa et al., 2015). Similarly, evidence suggests that poly-GA is also able to recruit poly-GR DPRs into cytoplasmic inclusions in *Drosophila*, HeLa cells, and cultured human neurons. Interestingly, the recruitment of poly-GR into inclusions by poly-GA DPRs seems to reduce poly-GR toxicity and restore Notch signaling in *Drosophila* (Yang et al., 2015). This study, together with the study by McEachin et al., supports Lee and colleagues’ proposal that poly-GA is a key mediator of cytotoxicity and interaction between DPRs. Indeed, poly-GA was found to be the most toxic DPR species in both *in vitro* and *in vivo* in the chick embryonic spinal cord. In *in vitro* and *in vivo* chick embryo models, poly-GP and poly-PA, but not poly-GR and poly-PR, are sequestered by poly-GA aggregates, and curiously, poly-PA seems to reduce poly-GA toxicity by inhibiting further aggregation of poly-GA (Lee et al., 2017). Altogether, evidence suggests that individual DPRs interact with each other and that this may affect disease pathology.

The advent of new studies unraveling the mechanisms of DPR translation has opened up the field of research regarding the possibility of previously unknown DPR species, as demonstrated by McEachin et al. (2020a) ascertaining the likely possibility of cDPRs. Identification of new DPR species implies that the composition of DPRs appears to be much more complicated than previously speculated. As the immediate sequence surrounding the repeat region can alter the behavior, localization, and toxicity of DPRs (He et al., 2020), future work should also consider both C and N-terminus amino acids to more accurately portray the role of each DPR in C9ORF72 ALS/FTD pathogenesis. Thus, to better understand how DPRs contribute to disease, future research should focus on disease models that express multiple DPRs, ideally at physiologically relevant levels. However, the development of such models still requires further advances in our current understanding of RAN translation to estimate the precise translated levels of different DPRs in patients.

## Author Contributions

AS, JP, IO, NM, and SS wrote the review. SS supervised the overall review. All authors contributed to the article and approved the submitted version.

## Conflict of Interest

The authors declare that the research was conducted in the absence of any commercial or financial relationships that could be construed as a potential conflict of interest.
